# Epidermolysis Bullosa Classification and Current Approach to Diagnosis

**DOI:** 10.1111/pde.70143

**Published:** 2026-09-04

**Authors:** Hannah E. Mumber, Marissa J. Perman

**Affiliations:** ^1^ Department of Dermatology Hospital of the University of Pennsylvania Philadelphia Pennsylvania USA; ^2^ Section of Dermatology Children's Hospital of Philadelphia Philadelphia Pennsylvania USA; ^3^ Department of Pediatrics Perelman School of Medicine at the University of Pennsylvania Philadelphia Pennsylvania USA

**Keywords:** classification, diagnosis, epidermolysis bullosa, genodermatoses, skin fragility

## Abstract

Epidermolysis bullosa (EB) is a heterogeneous group of rare genodermatoses marked by skin fragility and bullae formation induced by minor trauma. Pathologic variants in at least 21 genes are associated with EB, grouped into four major subtypes based predominantly on the plane of cleavage within the skin. EB simplex is characterized by epidermal bullae formation and is due to gene mutations that affect epidermal proteins, most commonly keratin filaments. Junctional EB is due to gene mutations affecting proteins in the basement membrane zone, causing a split within the lamina lucida of the dermal‐epidermal junction. Dystrophic EB is characterized by subepidermal bullae formation and is due to mutations in the gene encoding type VII collagen, which makes up the anchoring fibrils in the papillary dermis. Kindler EB is the rarest subtype and may be associated with cleavage at various levels within the skin due to a mutation in the *FERMT1* gene causing defects in kindlin‐1, a protein associated with integrins and focal adhesions. Because EB is such a heterogeneous disease, an understanding of genotype–phenotype correlations is necessary to help guide management. Traditionally, the first step in diagnosis was inducing a blister that was biopsied for immunofluorescence mapping. Currently, the gold standard for diagnosis is a blood sample or buccal swab for extraction of genomic DNA via next‐generation sequencing, which can identify the exact causative gene. A diagnosis of EB is life altering for patients and families alike. A firm understanding of EB classification and initial diagnostic workup can help dermatologists feel empowered to support and counsel families.

## 1 Introduction

Epidermolysis bullosa (EB) is a heterogeneous group of rare genetic dermatoses characterized by skin fragility and blister formation inducible by minimal trauma. EB is often referred to as “the worst disease you've never heard of” and “the butterfly disease” due to the high morbidity and mortality associated with EB and skin that seems as fragile as butterfly wings, respectively.

A broad phenotypic spectrum of EB has been described, with 34 subtypes grouped into four major categories based predominantly on the plane of cleavage within the skin [1]. To date, pathologic variants in at least 21 genes have been linked to EB, accounting for the broad phenotypic spectrum seen across the different EB subtypes [1]. All forms of EB have a significant impact on quality of life, and some types lead to premature mortality [2]. An understanding of EB classification and current approach to diagnosis is paramount to both dermatologists and pediatric dermatologists alike.

The latest EB Consensus Conference in 2020 introduced the concept of genetic conditions marked by skin fragility, of which they proposed EB to be the prototype and distinct from other “EB‐related” skin fragility conditions that lack significant blistering and are instead marked by erosions, peeling skin, hyperkeratosis, or connective tissue disease manifestations [2]. In EB, even slight mechanical trauma leads to blistering due to affected proteins at the dermal‐epidermal junction. Normally, skin integrity results from components that make up the keratin cytoskeleton and desmosomes in the epidermis and the hemidesmosomes, focal adhesions, anchoring filaments, and anchoring fibrils at the basement membrane zone [1]. However, in EB, genetic mutations leading to failure in one of these key structures lead to a compromised skin barrier and the manifestations of mucocutaneous fragility seen clinically.

The four major categories of EB are EB simplex (EBS), junctional EB (JEB), dystrophic EB (DEB), and Kindler EB (Tables 1, 2, 3, 4). In EBS, the blister cleavage occurs within the epidermis, most often due to defective keratin filaments, and healing usually occurs without scarring. In JEB, the skin separates in the lamina lucida of the dermal–epidermal junction, and blistering leads to atrophic scarring. In DEB, the blister forms in the papillary dermis below the basement membrane at the level of the anchoring fibrils, made up of type VII collagen, leading to scarring and milia (although milia are not limited to DEB). In Kindler EB, there is cleavage at varying levels due to mutations in the *FERMT1* gene causing defects in kindlin‐1, a protein associated with integrins and focal adhesions. This defect leads to fragile skin, poikiloderma, photosensitivity, and atrophy [3].

**TABLE 1:** Epidermolysis bullosa simplex.

EBS subtype	Inheritance	Gene mutation	Affected protein	Notable clinical manifestations
Localized (formerly Weber–Cockayne)	Autosomal dominant	KRT5, KRT14	Keratins 5 and 14	Blistering on palms and soles, 1/4 with oral blistering, focal keratoderma in adults
Intermediate (formerly Köbner)	Autosomal dominant	KRT 5, KRT14	Keratins 5 and 14	Generalized blistering that improves with age, variable mucosal involvement, focal keratoderma, 1/5 with nail involvement
Severe (formerly Dowling–Meara)	Autosomal dominant	KRT 5, KRT14	Keratins 5 and 14	Generalized blistering in neonate, improves with age, herpetiform blistering, mucosal blistering, may have natal teeth, nails may shed and regrow
With mottled pigmentation	Autosomal dominant	KRT5 > KRT14, exophilin‐5	Keratin 5 > keratin 14 and exophilin‐5	Reticulated hyperpigmentation, punctate keratoses, keratoderma
Migratory circinate erythema	Autosomal dominant	KRT5	Keratin 5	Small blisters arise on background of migratory, circinate erythema at sites of trauma and heal with post‐inflammatory hyperpigmentation
Intermediate with cardiomyopathy	Autosomal dominant	KLHL24	Kelch‐like member 24	Generalized blistering in infancy that improves with time but accompanied by the development of progressive dilated cardiomyopathy that may lead to premature death. Hair loss, keratoderma, nail dystrophy, and congenital aplasia cutis may be seen
Intermediate	Autosomal dominant or autosomal recessive	PLEC	Plectin	Generalized blistering, nail loss, and variable mucosal involvement
Intermediate or severe	Autosomal recessive	KRT 5, KRT14	Keratins 5 and 14	Generalized blistering in infancy that may NOT improve with age, as well as keratoderma, nail dystrophy, oral and genital blistering, anemia
Localized or intermediate	Autosomal recessive	DST or EXPH5 (aka SLAC2B)	Bullous pemphigoid antigen 230 (BP230 aka BPAG1e, dystonin) or exophilin‐5 (aka synaptotagmin‐like protein homolog lacking C2 domains b, Slac2‐b)	Relatively mild blistering that improves with age
Intermediate with muscular dystrophy	Autosomal recessive	PLEC	Plectin	Generalized blistering, nail loss, and variable mucosal involvement as well as progressive muscular dystrophy that may present from infancy to adulthood. May also have severe involvement of oral, laryngeal, and urethral mucosae
Severe with pyloric atresia	Autosomal recessive	PLEC	Plectin	Generalized blistering, nail loss, and variable mucosal involvement as well as pyloric atresia, anemia, ear anomalies, growth deficiency, joint contractures, severe congenital aplasia cutis
Localized with nephropathy	Autosomal recessive	CD151 (aka TSPAN24)	CD151 antigen (aka tetraspanin 24)	Blistering commonly localized to shins, mucosal blistering, nephropathy resulting in proteinuria, rarely epilepsy

**TABLE 2:** Junctional epidermolysis bullosa.

JEB subtype	Inheritance	Gene mutation	Affected protein	Notable clinical manifestations
Severe	Autosomal recessive	LAMA3, LAMB3, LAMC2 >> COL17A1	Laminin 332 >> type XVII collagen	Blisters that heal with atrophic scarring, periungual blistering and erythema, anonychia, hoarseness due to laryngeal involvement, perioral granulation tissue, dental enamel hypoplasia, anemia, failure to thrive. Mortality due to sepsis or respiratory compromise often before 2 years of age
Intermediate	Autosomal recessive	LAMA3, LAMB3, LAMC2, COL17A1	Laminin 332 or type XVII collagen	Less severe blistering and mucosal involvement compared to severe JEB. Granulation tissue is rare. Alopecia
With pyloric atresia	Autosomal recessive	ITGA6, ITGB4	Integrin α6β4	Mortality usually in infancy. Generalized blistering with atrophic scarring, anemia, dental enamel hypoplasia, nail dystrophy or anonychia, rudimentary ears. No granulation tissue. May have severe congenital aplasia cutis
Localized	Autosomal recessive	LAMA3, LAMB3, LAMC2, COL17A1, ITGA6, ITGB4, ITGA3	Laminin 332, type XVII collagen, integrin α6β4, integrin α3 subunit	Localized blistering without scarring and minimal mucosal involvement. No granulation tissue. Nail dystrophy or anonychia and dental enamel hypoplasia
Inversa	Autosomal recessive	LAMA3, LAMB3, LAMC2	Laminin 332	Blistering localized to intertriginous sites. Nail dystrophy, dental enamel hypoplasia
Late onset	Autosomal recessive	COL17A1	Type XVII collagen	Milder blistering usually with onset between ages 5 and 8. May have nail dystrophy and dental enamel hypoplasia. Extracutaneous involvement is rare
Laryngo‐onycho‐cutaneous syndrome	Autosomal recessive	LAMA3	Laminin α3A	Blistering with exuberant granulation tissue formation. Hoarse cry due to severe involvement of larynx, which is a major cause of mortality in childhood. Also may have nail dystrophy and conjunctival granulation tissue leading to symblepharon and vision loss
With interstitial lung disease and nephrotic syndrome	Autosomal recessive	ITGA3	Integrin α3 subunit	Blistering may be mild or severe. Respiratory distress in infancy with progressive interstitial lung disease, often leading to mortality. Nephrotic syndrome with progressive proteinuria and focal segmental glomerulosclerosis that may lead to renal failure

**TABLE 3:** Dystrophic epidermolysis bullosa.

DEB subtype	Inheritance	Gene mutation	Affected protein	Clinical manifestations
Intermediate	Autosomal dominant or autosomal recessive	COL7A1	Type VII collagen	AD: blistering predominantly localized to hands, knees, elbows, lower legs, scarring with milia, nail dystrophy common AR: Generalized blistering with scarring and milia, mild anemia, esophageal and anal strictures
Localized	Autosomal dominant or autosomal recessive	COL7A1	Type VII collagen	Blistering often localized to hands and feet. Nail dystrophy and anonychia is common. Extracutaneous involvement rare
Pruriginosa	Autosomal dominant or autosomal recessive	COL7A1	Type VII collagen	Blistering, scarring, and milia along with intense pruritus with secondary skin changes, such as lichenification and prurigo‐like lesions especially on shins. Onset often in adolescence or adulthood
Self‐improving	Autosomal dominant or autosomal recessive	COL7A1	Type VII collagen	Blistering is prominent in infancy and may include aplasia cutis with spontaneous improvement, sometimes only with persistent nail dystrophy
Severe	Autosomal recessive >> autosomal dominant AND recessive (compound heterozygosity)	COL7A1	Type VII collagen	Widespread blistering with scarring and milia, joint contractures, pseudosyndactyly, “mitten deformity,” “shawl sign,” anemia, osteoporosis, failure to thrive, dental caries, alopecia, aggressive squamous cell carcinomas lead to early mortality
Inversa	Autosomal recessive	COL7A1	Type VII collagen	Generalized blistering common in infancy which overtime becomes more localized affecting only intertriginous regions and mucosal surfaces

**TABLE 4:** Kindler epidermolysis bullosa.

Kindler EB	Inheritance	Gene mutation	Affected protein	Clinical manifestations
Kindler	Autosomal recessive	FERMT1 (aka KIND1)	Fermitin family homolog 1 (aka kindlin‐1)	Blistering in infancy predominantly at acral sites that improves with age, progressive poikiloderma, skin atrophy, keratoderma, pseudosyndactyly, photosensitivity. Increased risk of aggressive squamous cell carcinoma. Mucosal involvement is common with blistering, gingivitis, periodontitis, and esophageal and anal strictures

## 2 Types of Epidermolysis Bullosa

### 2.1 EBS

**FIGURE 1:**
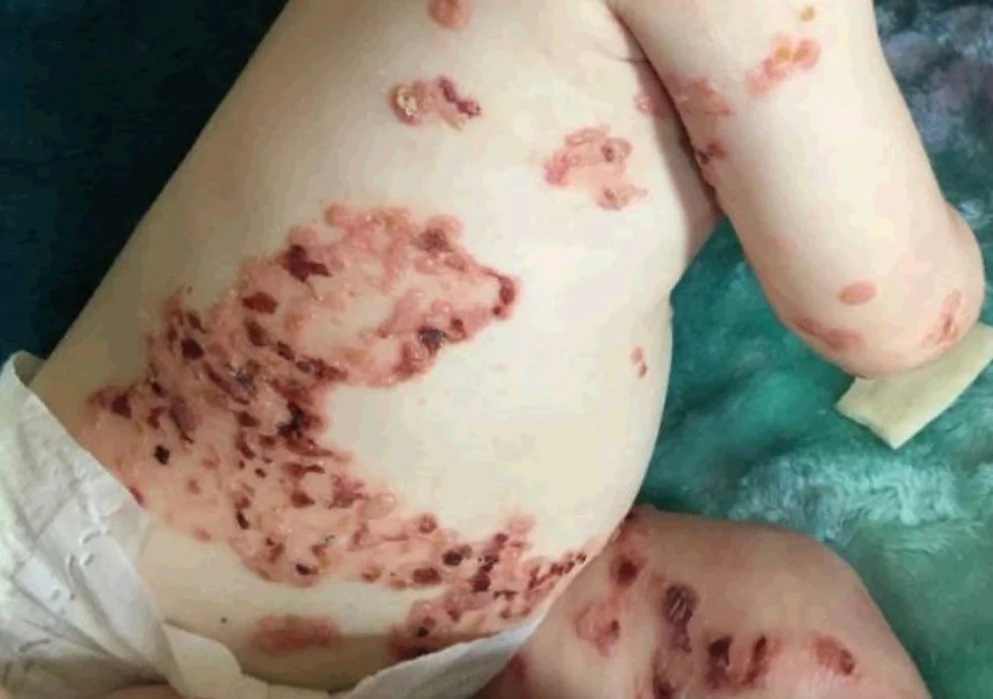
Herpetiform, grouped vesicles and hemorrhagically crusted pink plaques in a young patient with EBS due to a *keratin 5* mutation.

EBS is the most common subtype of EB and most often is inherited in an autosomal dominant manner. Localized EBS, previously known as Weber–Cockayne, presents with superficial blistering of the palms and soles. Many mild cases of EBS may go undiagnosed, but estimates of prevalence in the United States is 6 per million [4]. Intermediate EBS was previously known as generalized intermediate or Köbner. Severe EBS, previously known as generalized severe or Dowling–Meara, has more diffuse blistering in infancy that tends to become more clustered and herpetiform with advancing age (Figure 1) and is associated with protective hyperkeratosis particularly of palms and soles. Some subtypes, including severe EBS and those with extracutaneous involvement such as EBS caused by plectin mutations, can be fatal in infancy. EBS has a complex genetic framework with mutations identified in seven genes, which partially explains why treatment for EBS is lagging [2].

Autosomal dominant EBS is most often caused by mutations affecting keratin 5 (*KRT5*) and keratin 14 (*KRT14*). The phenotypic severity is determined by the position of the affected amino acid within the keratin polypeptide, with pathogenic variants affecting highly conserved amino acids within the helix initiation or termination motifs leading to severe EB while pathogenic variants in other areas of the gene lead to localized EBS [5]. More rarely, in‐frame deletions, splice site, or premature termination codon variants can lead to truncated proteins and severe phenotypes [6]. Recessive EBS is most often caused by *KRT14* nonsense or frameshift pathogenic variants [7].

Other rare subtypes of EBS include EBS with mottled pigmentation, EBS with migratory circinate erythema, localized or intermediate with BP230 deficiency or exophilin‐5 deficiency, intermediate with cardiomyopathy due to Kelch‐like member 24 (*KLHL24*) mutations, localized with nephropathy due to CD151 mutations, as well as intermediate with muscular dystrophy and severe with pyloric atresia, both of which are due to plectin mutations [2]. Mutations affecting plectin may result in either muscular dystrophy or pyloric atresia depending on the location of the pathogenic variant, with variants within exon 31 of *PLEC*, encoding the rod domain, being associated with muscular dystrophy while those outside exon 31 more commonly are associated with pyloric atresia [8].

In general, EBS tends to become milder over time. Patients with severe EBS tend to trend toward more localized EBS; those with localized EBS as children may no longer blister as adults but may continue to have nail dystrophy and hyperkeratosis. Oral blisters may present feeding difficulties in infancy, but this also tends to improve over time [3].

### 2.2 JEB

JEB is less common than both EBS and DEB, with an estimated incidence of about 2 per million live births, with lower prevalence often due to early mortality [9]. Severe blistering of the fingertips and nails in early infancy often clues into a diagnosis of JEB. The plane of cleavage that results in blistering in JEB is located in the lamina lucida of the basement membrane zone [3].

**FIGURE 2:**
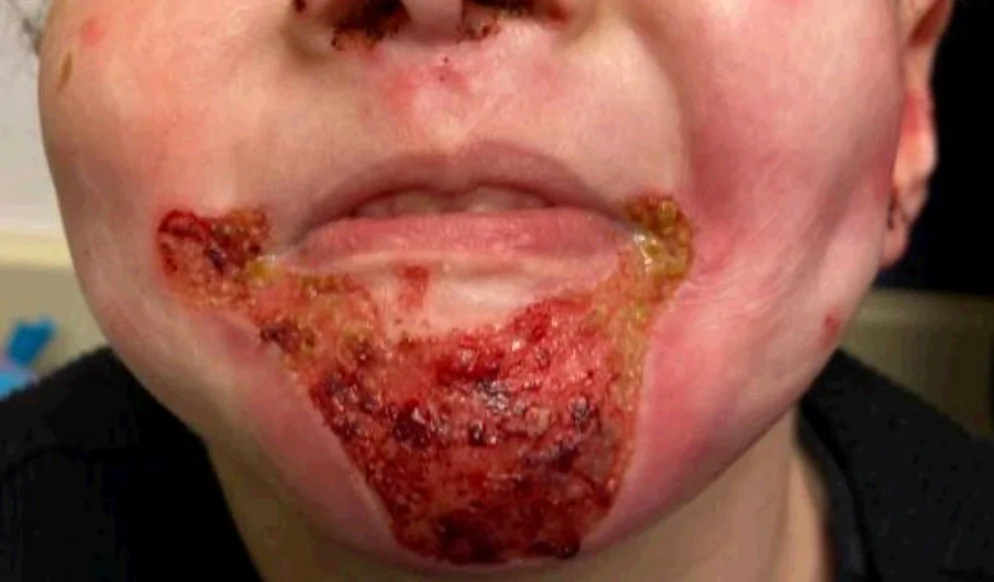
Prominent perioral granulation tissue in a patient with JEB due to a *LAMB3* mutation.

The two main subtypes of JEB are severe JEB, previously known as JEB generalized severe or Herlitz JEB, and intermediate JEB, previously known as JEB generalized intermediate or non‐Herlitz JEB. Bialleic mutations in the genes encoding the subunit chains of laminin 332 (*LAMA3*, *LAMB3*, and *LAMC2*), can result in either severe JEB or intermediate JEB [2]. Prominent granulation tissue of the perioral region is characteristic of JEB caused by the *LAMB3* mutation (Figure 2). Death from sepsis or respiratory compromise due to airway involvement commonly occurs before age 2 years in the severe generalized form of junctional EB, most often due to pathogenic variants causing absence of laminin 332 or integrin α6β4 [10]. Infants with severe JEB due to laminin 332 mutations often demonstrate a hoarse cry due to laryngeal involvement; they may have either minimal or widespread blistering, and often exhibit poor feeding and growth in part due to oral mucosal involvement. Patients with JEB also universally have dental anomalies such as generalized enamel hypoplasia and amelogenesis imperfecta that predispose to dental caries [11]. Biallelic mutations in type XVII collagen gene, *COL17A1*, more often result in intermediate JEB but may also rarely result in severe JEB. Most *COL17A1* pathogenic variants cause absence of collagen XVII but have less severe phenotypes [12].

Other rarer subtypes of JEB include JEB with pyloric atresia caused by defective integrin α6β4, localized JEB (caused by laminin 332, type XVII collagen, integrin α6β4, or integrin α3 subunit), JEB inversa caused by laminin 332, late onset JEB due to type XVII collagen, laryngo‐onycho‐cutaneous syndrome due to laminin α3A, and JEB with interstitial lung disease and nephrotic syndrome due to integrin α3 subunit [2]. Pathogenic variants in *COL17A1*, *LAMB3*, *LAMA3*, *LAMC2*, and *ITGB4* associated with self‐improving JEB and mild phenotypes may be due to alternative splicing or spontaneous read‐through or skipping of exons containing premature termination codons [13]. Individuals with splice site mutations or compound heterozygous mutations that enable low‐level expression of collagen XVII exhibit mild, self‐improving disease. For laminin‐332 genes, milder disease is often due to splice site or missense mutations. Rarely, missense or heterozygous mutations in *ITGB4* can cause late‐onset or mild skin‐limited JEB [14].

### 2.3 DEB

Dystrophic types of EB are divided into dominant and recessive forms, with RDEB generally being more severe. However, there may be phenotypic overlap between dominant and recessive forms. Estimated prevalence of DEB in the United States is 6 per million in the USA, with an incidence of 2.5 per million live births for DDEB and 3.05 per million for RDEB [9, 15].

**FIGURE 3:**
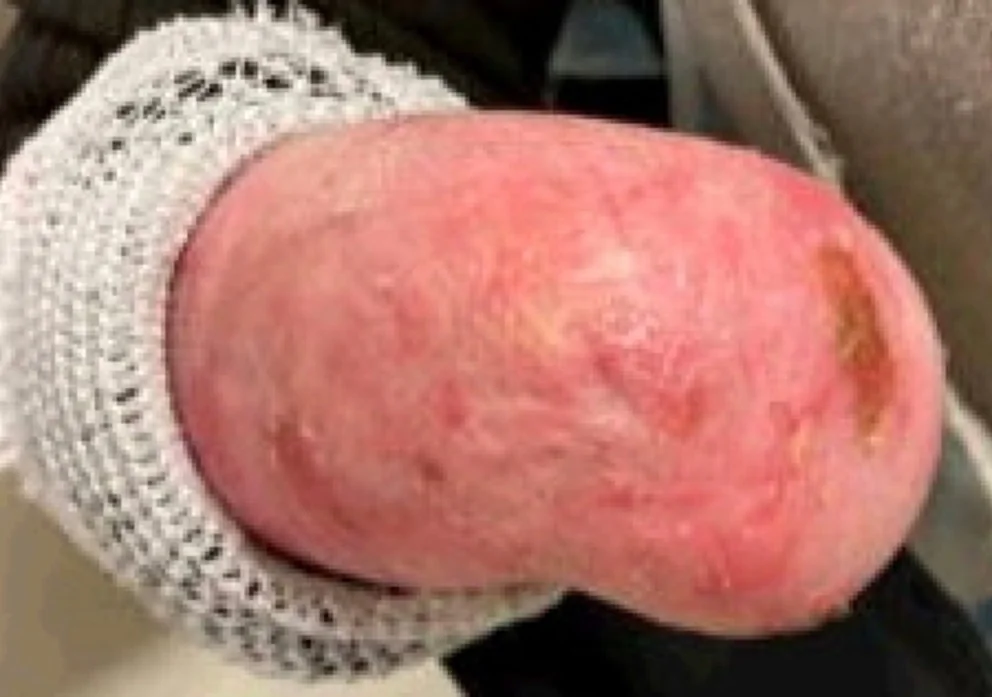
A patient with RDEB with severe “mitten deformity” of the hand due to chronic blistering, inflammation and fibrosis leading to pseudosyndactyl.

**FIGURE 4:**
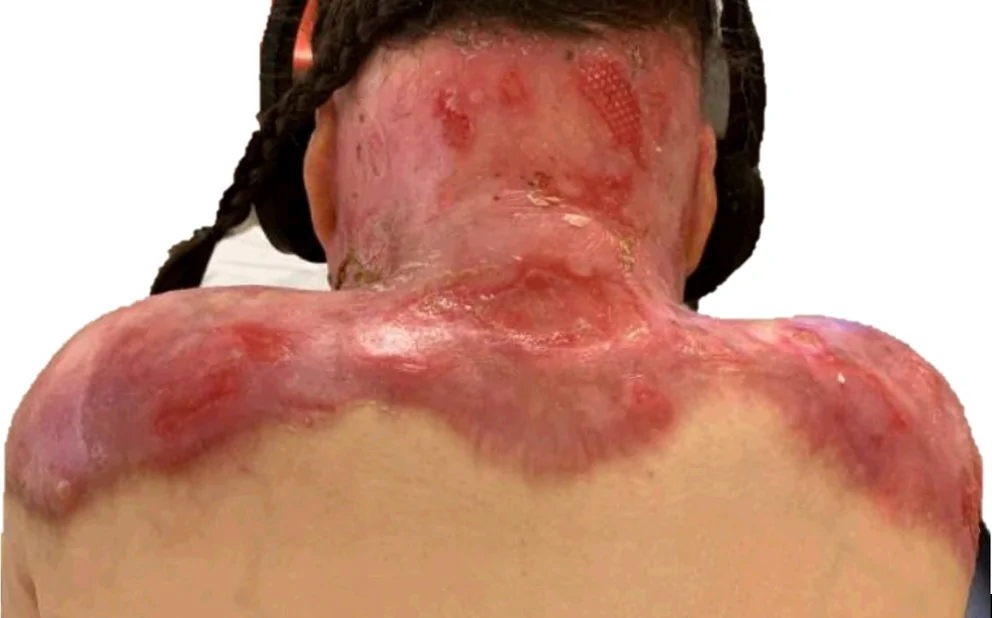
Characteristic “shawl sign” with large, confluent chronic erosions of the neck and upper back in patient with RDEB.

The plane of cleavage in DEB is in the superficial dermis just beneath the lamina densa due to defective type VII collagen, or *COL7A1*, which is the major component of the anchoring fibrils. Both lead to scarring following blistering in both the skin and mucosae, as well as milia formation in areas of healed blistering. In general, the dominant forms are less severe; affected individuals are usually of normal stature, have limited skin blistering, and predominantly have findings of nail dystrophy [3]. The major subtypes of DEB are localized DDEB, which includes nails only, pretibial, and acral DDEB, intermediate DDEB (previously known as generalized DDEB), intermediate RDEB (previously known as RDEB generalized intermediate or non‐Hallopeau–Siemens type RDEB), and severe RDEB (previously known as RDEB generalized severe or Hallopeau–Siemens type RDEB) [2]. Intermediate RDEB may affect the hands, feet, and legs more mildly than severe RDEB and also have nail dystrophy. Severe RDEB is characterized by severe, widespread blistering, the classic pseudosyndactyly known as the mitten deformity (Figure 3) and the “shawl sign,” (Figure 4) which is a characteristic chronic open wound along the upper back and posterior neck [3].

DEB typically presents with systemic involvement affecting multiple organs including the gastrointestinal tract, musculoskeletal system, genitourinary tract, eyes, and heart. Oral blistering, microstomia, and esophageal strictures lead to poor oral intake and compromised nutrition, contributing to anemia and failure to thrive [11]. Patients may suffer from both urethral and anal strictures. Joint contractures and hand deformities limit mobility and function. Corneal erosions and scarring may lead to visual impairment. Cardiomyopathy has been associated with severe forms of DEB. Non‐healing wounds, frequent infections with staphylococcus, streptococcus, and pseudomonas, as well as chronic inflammation often set the stage for the development of aggressive cutaneous squamous cell carcinoma, which is the leading cause of death in RDEB [3, 16]. Given the severity often associated with DEB, there is significant psychosocial impact with high rates of anxiety, depression, obsessive‐compulsive disorder, and familial stress [17].

Other rarer variants within localized DDEB include pruriginosa and self‐improving and within intermediate RDEB include inversa, localized, pruriginosa, and self‐improving [2].

DDEB is most commonly due to glycine substitutions around the hinge region of type VII collagen corresponding to exon 73 of *COL7A1*, although the same glycine substitution can still result in variable phenotypes [18]. Splice‐site or indel mutations that lead to in‐frame skipping of exons or deletions within the triple‐helical domain lead to mild localized DDEB [19]. Many different pathogenic variants result in the absence of type VII collagen and thus RDEB [2]. Compound heterozygosity for dominant and recessive *COL7A1* mutations results in severe DEB [20]. Specific glycine substitutions and in‐frame skipping of exons have been implicated in self‐improving DEB, also known as transient bullous dermolysis of the newborn [21]. RDEB inversa is caused by specific glycine and arginine substitution mutations in *COL7A1* [22].

### 2.4 Kindler EB

Kindler EB is the rarest subtype of EB, with only about 250 reported cases since its description in 1954 and is distinguished by poikiloderma and photosensitivity in addition to skin fragility and blistering [23]. Kindler EB is caused by mutations in *FERMT1*, also known as *KIND1*, which encodes fermitin family homolog 1, also known as kindlin‐1, an intracellular protein involved in focal adhesions [3]. In addition to skin fragility, patients with Kindler EB may have fragility of multiple mucosal surfaces, including the oral, ocular, gastrointestinal, and genitourinary mucosae [11].

### 2.5 Syndromic EB

Syndromic types of EB are defined by those whose affected genes also have roles in extracutaneous tissues, therefore resulting in extracutaneous organ system involvement [24]. The most common examples are muscular dystrophy in patients with EBS caused by plectin deficiency, pyloric atresia in patients with EBS with plectin deficiency and in patients with JEB with integrin α6β4 deficiency, cardiomyopathy in patients with EBS caused by *KLHL24* or *PLEC* variants and in skin fragility syndromes with desmoplakin (*DSP*) and junction plakoglobin (*JUP*) mutations, lung fibrosis and nephrotic syndrome in patients with JEB due to deficiency of the integrin α3 subunit, connective tissue issues in patients with *PLOD3* gene mutations, and nephrotic syndrome in patients with CD151 deficiency [25, 26, 27, 28].

### 2.6 Other EB‐Related Conditions

Other inherited diseases with skin fragility but without predominant skin blistering are considered EB‐related conditions rather than classical EB subtypes and should be on the differential diagnosis when considering the diagnosis of EB in a newborn. Thus, their implicative genes are included in next‐generation sequencing (NGS) targeted panels for EB. Examples include acral peeling skin disorders (caused by *TGM5* targeting transglutaminase 5, *CSTA* targeting cystatin A, *CTSB* targeting cathepsin B, *SERPINB8* targeting serpin protease inhibitor 8, *FLG2* targeting filaggrin 2, *CDSN* targeting corneodesmosin, *CAST* targeting calpastatin, *DSG1* targeting desmoglein 1, *SPINK5* targeting LEKT1), erosive disorders with acantholysis due to desmosomal defects (caused by *DSP* targeting desmoplakin, *JUP* targeting plakoglobin, *PKP1* targeting plakophilin 1, *DSC3* targeting desmocollin 3, *DSG3* targeting desmoglein 3), keratinopathic ichthyoses that exhibit blistering in infancy but later predominated by hyperkeratosis (*KRT1*, *KRT10*, *KRT2* targeting keratins 1, 10, and 2, respectively), pachyonychia congenita (*KRT6A*, *KRT6B*, *KRT6C*, *KRT16*, *KRT17* targeting keratins 6A, 6B, 6C, 16, and 17, respectively), and syndromic connective tissue disorder with skin fragility (*PLOD3* targeting lysyl hydroxylase 3) [2].

## 3 Diagnosis of Epidermolysis Bullosa

Any newborn demonstrating signs of skin blistering or fragility should be referred to an EB center as soon as possible. Early diagnosis and classification of EB along with familiarity with management helps improve clinical outcomes by equipping families with knowledge on prognosis, management options, potential clinical trials, and genetic counseling opportunities [29]. Referral to the Dystrophic Epidermolysis Bullosa Research Association (DEBRA) patient group, which provides care and support for patients and families with all types of EB, is essential to help families cope with the diagnosis and receive support and dressing supplies. DEBRA also provides resources for healthcare providers.

A diagnosis of EB can be suspected from clinical presentation and family history. However, it is often impossible to predict EB subtype from clinical presentation at birth alone. EB may be caused by a variety of different gene mutations, as evidenced above, with both instances of dominant and recessive inheritance. In instances of EB diagnosis with apparent de novo occurrence without a family history, genetic testing is necessary to differentiate between autosomal dominant and recessive inheritance by testing both the patient and parents [30].

Skin biopsy of a blister for immunofluorescence mapping (IFM) was traditionally the first step in the diagnosis of suspected EB. Currently, the gold standard for diagnosis is blood sample or buccal swab for extraction of genomic DNA via NGS, which can identify the exact causative gene [1]. NGS uses high‐throughput technology to sequence either targeted gene panels or entire exomes, genomes, or transcriptomes [31]. DEBRA International developed clinical practice guidelines in 2016 for the laboratory diagnosis of EB. Their recommendations continue to include skin biopsy in addition to DNA testing in order to allow for complete molecular characterization at both the DNA and protein levels, ultimately allowing for a more thorough prediction of genotype–phenotype correlations [1, 32].

While advances in genetic testing have made definite diagnosis possible and turnaround time continues to decrease, IFM can help provide a diagnosis within hours and was traditionally less costly than genetic testing, often making it the diagnostic method of choice in resource‐poor settings. In addition to its utility for IFM, skin biopsy can also aid in diagnosis by allowing for skin ultrastructure examination by transmission electron microscopy (TEM) [1, 32]. However, a biopsy, especially in newborns, can be traumatic and challenging as a new blister must be induced by twisting a pencil eraser against intact skin. Currently, in the United States, most experienced EB providers choose to use NGS via blood or saliva sample as soon as EB is suspected [33].

Sanger sequencing (SS) is a straightforward approach that is time and cost‐effective for gene identification in straightforward cases or when the familial mutation is already known. Both NSG and SS approaches are able to identify the disease‐causing variant in most cases of EB [1]. However, given advances with NSG, SS is now much less commonly used.

Targeted NSG with known EB genes is advantageous in that it has higher coverage per gene and base. Depending on the panel and laboratory, NGS panels for EB may include anywhere from 11 to 33 genes associated with skin fragility [1, 31]. Whole exome sequencing (WES) and whole genome sequencing (WGS) are powerful tools in their ability to identify new EB‐causative genes.

Rarely, skin fragility may be so mild that diagnosis is not suspected until later in childhood or adulthood. In this case, the patient should be referred to an EB center to arrange for genetic testing.

Once a causative variant is identified for a patient, their biological parents and siblings should undergo genetic testing to assess for carrier status. This helps with understanding the inheritance pattern and is helpful for determining the pathogenicity of variants of uncertain significance (VUS). Genetic counseling often includes genetic testing of the partner of carriers in the family. Referral for prenatal testing by linkage analysis is often an option [1].

If no disease‐causing variant is found with SS or NSG with an EB gene panel in a patient with high suspicion for EB based on clinical presentation or IFM or TEM findings, other diagnostic techniques such as multiplex ligation‐dependent probe amplification, reverse transcriptase PCR, RNA‐sequencing, or Western blotting should be pursued in order to try to obtain evidence of alternative chromosomal rearrangements or gene expression alterations [1, 19]. If workup is still negative, open‐exome analysis may be done [1].

## 4 Conclusion

A diagnosis of EB is life altering for patients and families alike. Dermatologists can help alleviate some of the stress associated with a new diagnosis of EB by guiding families toward appropriate resources and support. A firm understanding of EB classification and initial diagnostic workup can help dermatologists feel empowered to support and counsel families during the diagnosis period.

## Conflicts of Interest

Marissa J. Perman has consulted for Abeona Therapeutics and Chiesi USA. She has been a speaker for Chiesi USA. She is a PI for Aegle Therapeutics, Chiesi USA, and Abeona Therapeutics. The other author declares no conflicts of interest.

## Data Availability

Data sharing not applicable to this article as no datasets were generated or analysed during the current study.
